# Nrf2 Activation Sensitizes K-Ras Mutant Pancreatic Cancer Cells to Glutaminase Inhibition

**DOI:** 10.3390/ijms22041870

**Published:** 2021-02-14

**Authors:** Shin Hamada, Ryotaro Matsumoto, Yu Tanaka, Keiko Taguchi, Masayuki Yamamoto, Atsushi Masamune

**Affiliations:** 1 Division of Gastroenterology, Tohoku University Graduate School of Medicine, 1-1 Seiryo-machi, Aoba-ku, Sendai, Miyagi 980-8574, Japan; rmat44@gmail.com (R.M.); y.tanaka1055@gmail.com (Y.T.); amasamune@med.tohoku.ac.jp (A.M.); 2 Department of Medical Biochemistry, Tohoku University Graduate School of Medicine, 2-1 Seiryo-machi, Aoba-ku, Sendai, Miyagi 980-8575, Japan; keiko@med.tohoku.ac.jp (K.T.); masiyamamoto@med.tohoku.ac.jp (M.Y.)

**Keywords:** Keap1, Nrf2, glutaminase, CB-839, BPTES

## Abstract

Pancreatic cancer remains intractable owing to the lack of effective therapy for unresectable cases. Activating mutations of *K-ras* are frequently found in pancreatic cancers, but these have not yet been targeted by cancer therapies. The Keap1-Nrf2 system plays a crucial role in mediating the oxidative stress response, which also contributes to cancer progression. Nrf2 activation reprograms the metabolic profile to promote the proliferation of cancer cells. A recent report suggested that *K-ras*- and Nrf2-active lung cancer cells are sensitive to glutamine depletion. This finding led to the recognition of glutaminase inhibitors as novel anticancer agents. In the current study, we used murine pancreatic cancer tissues driven by mutant *K-ras* and *p53* to establish cell lines expressing constitutively activated Nrf2. Genetic or pharmacological Nrf2 activation in cells via *Keap1* deletion or Nrf2 activation sensitized cells to glutaminase inhibition. This phenomenon was confirmed to be dependent on K-ras activation in human pancreatic cancer cell lines harboring mutant *K-ras*, i.e., Panc-1 and MiaPaCa-2 in response to DEM pretreatment. This phenomenon was not observed in BxPC3 cells harboring wildtype *K-ras*. These results indicate the possibility of employing Nrf2 activation and glutaminase inhibition as novel therapeutic interventions for *K-ras* mutant pancreatic cancers.

## 1. Introduction

The prognosis of pancreatic cancer remains dismal despite improved diagnostic methods. The main reason for this is the lack of effective therapies for advanced pancreatic cancer. Targeted therapies for driver mutations in several types of malignancies such as gastrointestinal stromal tumor and non-small-cell lung cancer with *KIT* mutation and *ALK* translocation, respectively, have led to better prognosis [1,2]. However, more than 90% of the pancreatic cancers harbor activating *K-ras* mutations [3], but these mutations have not yet been successfully targeted. The molecules downstream of K-ras signaling are recognized as alternative targets, such as mitogen-activated protein/extracellular signal-regulated kinase kinase and [4] and protein kinase C [5].

The Keap1-Nrf2 system is pivotal in the maintenance of normal tissue structure and organ protection from oxidative stress. Conformational changes in Keap1 induced by cellular reactive oxygen species and electrophiles result in the nuclear accumulation of Nrf2, a transcription factor that induces the expression of cytoprotective genes [6]. The deletion of *Nrf2* in mouse models with mutant *K-ras‒*driven pancreatic cancer resulted in reduced formation of pancreatic lesions [7,8]. A few lung and esophageal cancers harbor loss-of-function mutations—in *Keap1*—or degradation-resistant mutations—in *Nrf2*—resulting in the constitutive stabilization of Nrf2 [9,10]. Direct inhibition of Nrf2 by a small molecule inhibitor, halofuginone, reportedly suppressed the growth of cancer cells with constitutive activation of Nrf2 both in vitro and in vivo [11].

Activation of Nrf2 affects cellular metabolism. The aberrant accumulation of p62/Sequestosome 1 (Sqstm1) stabilizes Nrf2 in hepatocellular carcinoma. This stabilization reprograms glucose and glutamine metabolism, and results in the development of resistance against chemotherapeutic agents [12]. Similarly, loss of *Keap1* promotes activating mutant *K-ras*-driven, glutaminolysis-dependent lung cancer development [13]. Several glutaminase inhibitors have shown antitumor activity in these glutaminolysis-dependent cancer cells. These include telaglenastat (CB-839) [14] and bis-2-(5-phenylacetamido-1,3,4-thiadiazol-2-yl) ethyl sulfide (BPTES) [13]. The antitumor activity of CB-839 is potentiated upon combining with conventional chemotherapeutic agents [15] and vice versa [16], suggesting the possibility of glutaminase inhibition as a novel therapeutic strategy.

To clarify whether Nrf2 activation has similar effects in pancreatic cancer, we established mouse pancreatic cancer cell lines harboring *Keap1* deletion, which resulted in the constitutive activation of Nrf2. These cell lines were more susceptible to glutaminase inhibitors than cell lines lacking *Keap1* and *Nrf2*. Furthermore, pretreatment of mutant *K-ras* expressing mouse and human pancreatic cancer cell lines with diethyl malate (DEM), an electrophilic stress inducer, sensitized the cells to a glutaminase inhibitor. These data suggest that the combination of an Nrf2 activator and a glutaminase inhibitor might serve as an effective therapeutic approach for pancreatic cancer.

## 2. Results

### 2.1. Establishment of Cell Lines Expressing Constitutively Activated Nrf2

**Figure 1:**
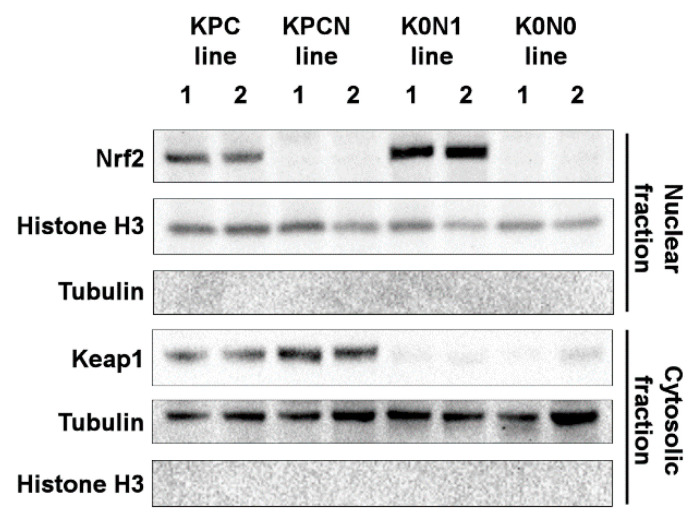
Expression of Nrf2 and Keap1 in KPC, KPCN, K0N1, and K0N0 lines. Histone H3 and tubulin were used as loading controls for the proteins present in nuclear and cytosolic fractions, respectively.

*Pdx-1-Cre::K-ras^LSL-G12D/+^::p53^LSL-R172H/+^::Keap1^fl/fl^::Nrf2^+/−^* (KPC::K0N1) and *Pdx-1-Cre::K-ras^LSL-G12D/+^::p53^LSL-R172H/+^::Keap1^fl/fl^::Nrf2^−/−-^* (KPC::K0N0) mice developed invasive pancreatic cancers (2/31 and 2/17 mice, respectively) within 90 days of birth. We established cell lines expressing constitutively activated Nrf2 or with *Keap1*/*Nrf2* deletion from these pancreatic cancer tissues using a pre-established protocol [8]. As shown in Figure 1, KPC::K0N1-mice‒derived cell lines (K0N1 lines 1 and 2) displayed increased nuclear accumulation of Nrf2 compared with KPC-mice‒derived pancreatic cancer cell lines (KPC lines 1 and 2). KPC::Nrf2^−/−^-mice‒derived pancreatic cancer cell lines (KPCN lines 1 and 2) lack Nrf2, and KPC::K0N0 mice-derived cell lines (K0N0 line 1 and 2) lacked both Nrf2 and Keap1 expression.

### 2.2. Increased Expression of Nrf2-Target Genes in Cell Lines Expressing Constitutively Activated Nrf2

**Figure 2:**
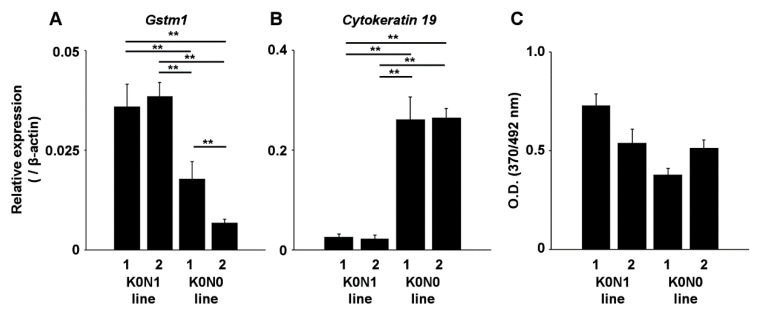
Real-time RT-PCR for checking the expression of *Gstm1* (**A**) and *Cytokeratin 19* (**B**) in K0N1 and K0N0 cell lines (*N* = 4). ** indicates *p* < 0.01 by the Tukey–Kramer method. (**C**) BrdU assay in K0N1 and K0N0 cell lines following culture for 24 h in normal growth medium (*N* = 6). The error bars show standard deviations.

To confirm the transcriptional activity of Nrf2, we assessed the expression of an Nrf2-target gene, *glutathione S-transferase M1* (*Gstm1*) and a cytoskeletal gene *cytokeratin 19* in the K0N1 and K0N0 lines. The K0N1 cell lines exhibited higher expression of *Gstm1* compared with K0N0 lines (Figure 2A), suggesting increased transcriptional activity of Nrf2. In contrast, K0N1 cell lines exhibited lower expression of *cytokeratin 19* compared with K0N0 lines (Figure 2B). These findings indicated that constitutive activation of Nrf2 had an impact on the epithelial phenotype of cancer cells. The proliferation of K0N1 cell lines was not significantly different from that of K0N0 lines, i.e., the variability between lines did not affect the proliferation (Figure 2C). We also assessed tumorigenicity by subcutaneous implantation of these cells in nude mice. Transplantation of K0N1 cell line 1 resulted in the development of subcutaneous tumors, which were similar in size to those formed upon the transplantation of K0N0 line 1 (supplemental Figure S1). We confirmed that Nrf2 was activated in cell lines derived from KPC::K0N1 tumors. However, proliferation was not dependent on Nrf2 levels in cell lines derived from KPC tumors.

### 2.3. Cell Lines Expressing Constitutively Activated Nrf2 Are Sensitive to Glutaminase Inhibitors

**Figure 3:**
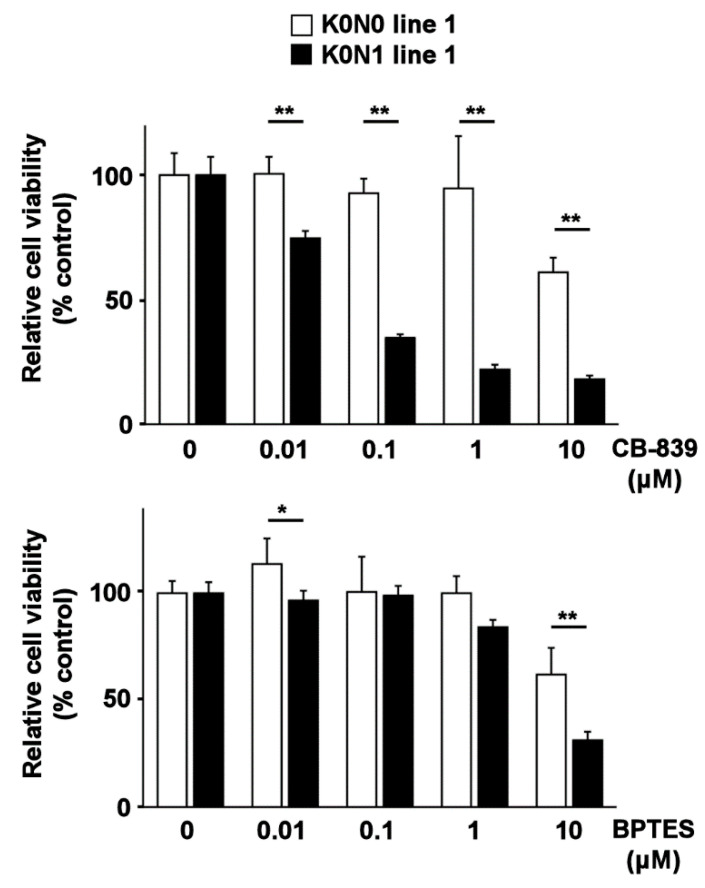
MTT viability assay for K0N1 and K0N0 cell lines after treatment with CB-839 (upper panel) and BPTES (lower panel) (*N* = 6, 5000 cells/well, 48 h). * and ** indicate *p* < 0.05 and *p* < 0.01 by the Tukey–Kramer method, respectively. The error bars show standard deviations.

Next, we treated K0N0 and K0N1 cell lines with the glutaminase inhibitors CB-839 and BPTES. Both inhibitors significantly decreased the viability of K0N1 line 1 compared to that of K0N0 line 1 (Figure 3). The K0N1 line 2 was equally sensitive to CB-839 and BPTES. These results indicated that the glutaminase is responsible for the viability of K0N1 cell lines.

### 2.4. Nrf2 Inducer Sensitizes Murine Pancreatic Cancer Cell Lines to Glutaminase Inhibitor

**Figure 4:**
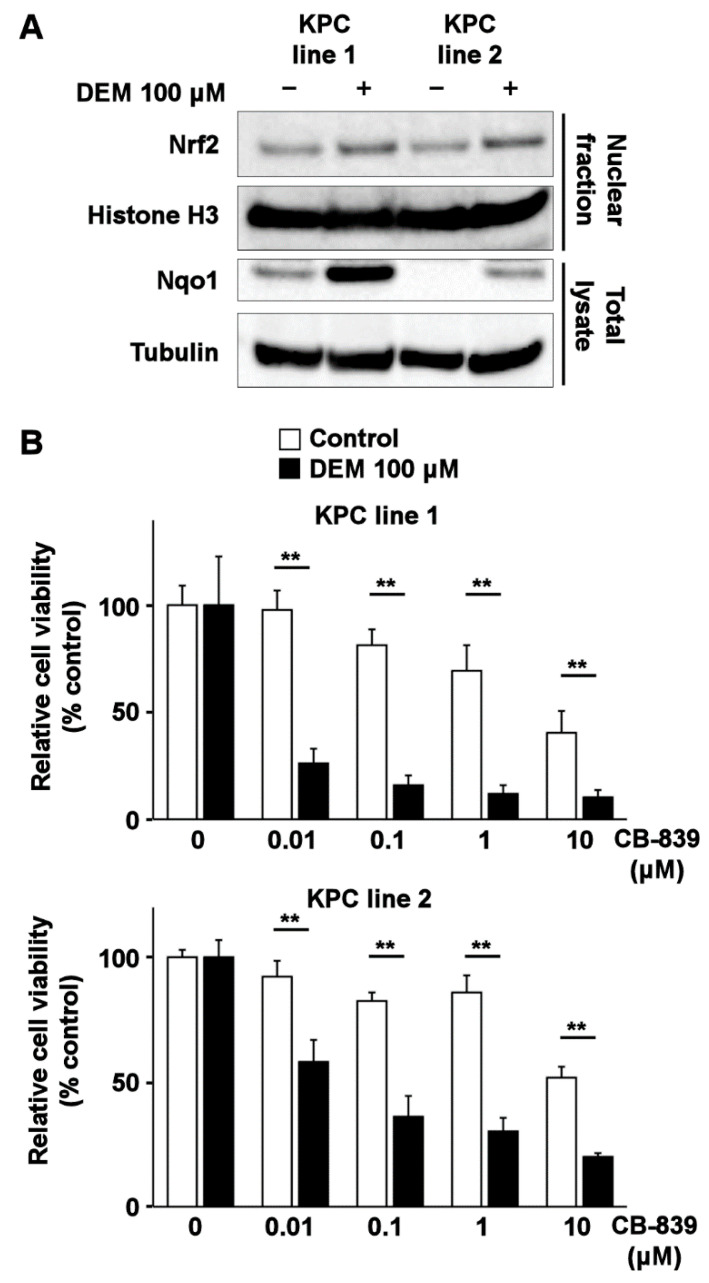
Induction of Nrf2 and sensitization to CB-839 by DEM treatment in KPC lines. (**A**) Nuclear accumulation of Nrf2 and induction of Nqo1 expression in KPC cell lines after DEM treatment. Histone H3 and tubulin are displayed as loading controls for proteins present in the nuclear and cytosolic fractions, respectively. (**B**) MTT assay for analyzing the viability of KPC cell lines after CB-839 treatment (*N* = 6, 5000 cells/well, 48 h; with or without DEM pretreatment (100 μM, 24 h)). ** indicates *p* < 0.01 by the Tukey–Kramer method. The error bars show standard deviations.

To confirm that oxidative stress-induced activation of Nrf2 was sufficient to sensitize cancer cells to glutaminase inhibition, we treated KPC cell lines with the Nrf2 inducer, DEM. Treatment of KPC lines with DEM resulted in increased Nqo1 expression and nuclear accumulation of Nrf2, a hallmark of the oxidative stress response (Figure 4A). Treatment of KPC cell lines with CB-839 in the background of DEM reduced the viability of these cells (Figure 4B), suggesting that induction of Nrf2 activation sensitized KPC cancer cells to glutaminase inhibition.

### 2.5. Nrf2 Inducer Sensitizes Human Pancreatic Cancer Cell Lines Harboring K-Ras Mutation to Glutaminase Inhibitor

**Figure 5:**
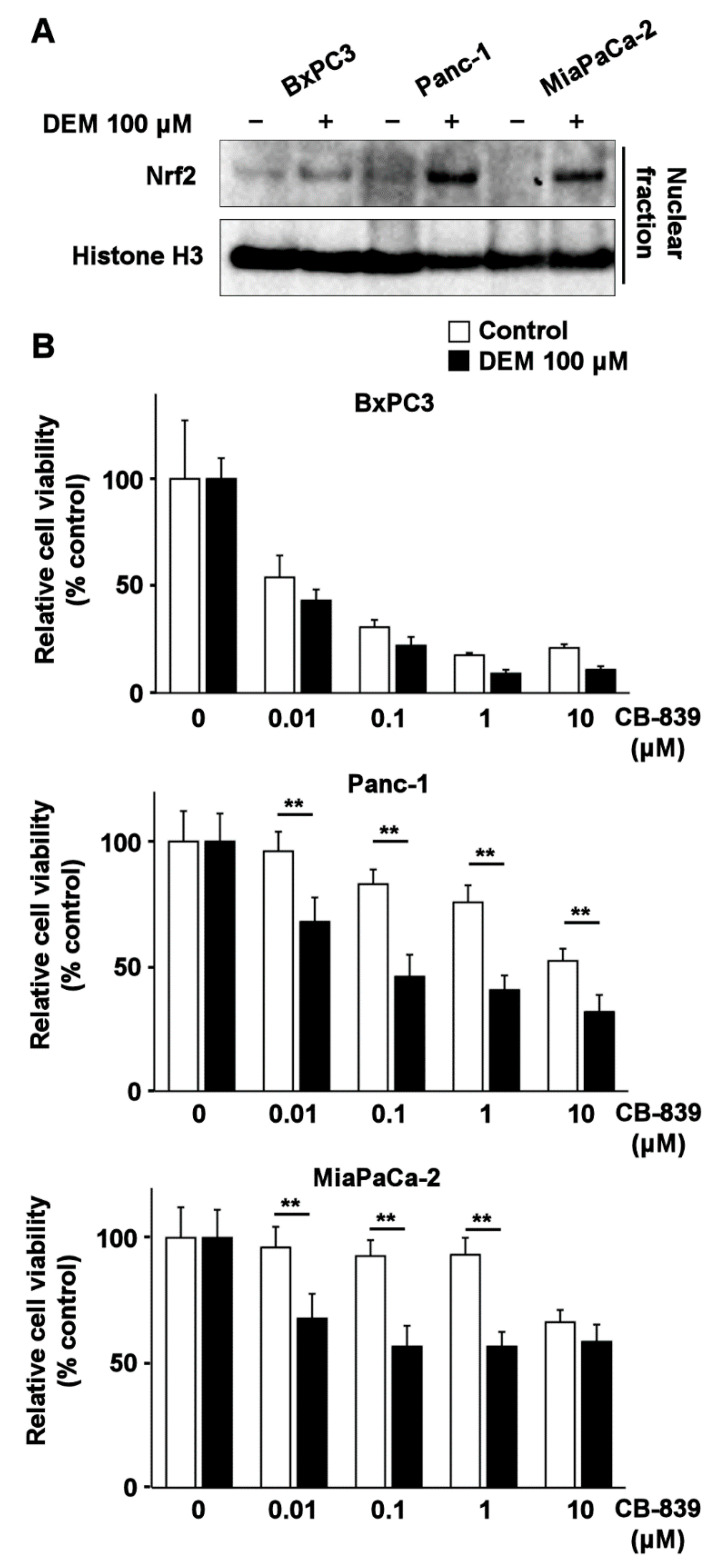
Induction of Nrf2 and sensitization to CB-839 by DEM treatment in human pancreatic cancer cell lines. (**A**) Nuclear accumulation of Nrf2 in BxPC3, Panc-1, and MiaPaCa-2 cells after DEM treatment. Histone H3 is used as the loading control. (**B**) MTT assay for checking the viability of BxPC3, Panc-1 and MiaPaCa-2 after CB-839 treatment (*N* = 6, 5000 cells/well, 48 h; with or without DEM pretreatment (100 μM, 24 h)). ** indicates *p* < 0.01 by the Tukey–Kramer method. The error bars show standard deviations.

To further validate these results, we examined whether DEM enhances the inhibitory effect of CB-839 on the viability of human pancreatic cancer cell lines. BxPC3 cells—harboring *K-ras* wildtype—Panc-1 and MiaPaCa-2 cells—harboring mutant *K-ras* [17]—exhibited nuclear accumulation of Nrf2 following DEM treatment (Figure 5A). Treatment of Panc-1 and MiaPaCa-2 cells with CB-839 in the background of DEM treatment resulted in reduced cell viability (Figure 5B); however, this phenomenon was not observed in BxPC3 cells. These results indicated that the glutaminase inhibitor CB-839 effectively reduced the viability of human and mouse cell lines exhibiting K-ras activation.

## 3. Discussion

In this study, we observed increased sensitivity to glutaminase inhibition in pancreatic cancer cell lines harboring the active forms of K-ras and Nrf2. In addition to the constitutive activation of Nrf2 by *Keap1* deletion, DEM-induced Nrf2 activation was sufficient to sensitize cancer cells to glutaminase inhibition. In a previous study, the Nrf2-activating effects of mutant *K-ras* had been demonstrated in a KPC mouse model [7]. Mouse-derived cancer cell lines in this study were mutant *K-ras* driven, therefore we assume the basal Nrf2 activity might be increased. The pancreatic cancer cell line BxPC3—harboring *wildtype K-ras*—could not be further sensitized to glutaminase inhibition. Nrf2 activation by mutant K-ras signaling might be necessary for the induction of glutamine dependence.

Interestingly, constitutive activation of Nrf2 in the K0N1 cell lines did not induce tumorigenicity in vivo. We had previously reported that pancreas-specific mutant *K-ras* expression and *Keap1* deletion resulted in progressive pancreatic atrophy [18]. This phenotype was rescued upon the heterozygous deletion of Nrf2, suggesting that a certain threshold of Nrf2 expression is required for pancreatic atrophy. Indeed, *Pdx-1-Cre::K-ras^LSL-G12D/+^::p53^LSL-R172H/+^::Keap1^fl/fl^::Nrf2^+/+^* mice (KPC::Keap1 CKO mice) also exhibited pancreatic atrophy (but no pancreatic tumors; unpublished data). These results with the current observations suggest that excess Nrf2 activation might be associated with certain disadvantages in pancreatic lineage cells. In contrast to that in KPC::Keap1 CKO mice, *Keap1* deletion in mice expressing liver-specific mutant *K-ras* and *p53* resulted in accelerated cholangiocarcinoma development [19]. Therefore, the existence of mutant *K-ras* and activation of Nrf2 might suppress the unknown machinery that is indispensable for cell survival in pancreatic cancer. The organ-specific role of Nrf2 activation might be a specific target in pancreatic cancer.

Glutamine deprivation in pancreatic cancer cells has recently attracted attention as a therapeutic strategy [20]. The current study provides a novel method to sensitize pancreatic cancer cells to glutamine deprivation by activating Nrf2. As the Keap1-Nrf2 pathway plays an important role in energy metabolism, further studies are warranted to clarify the mechanism underlying its antitumor effects. The similar study has been reported in lung cancer. In *K-ras* mutant lung cancer, Nrf2 also contributes to glutamine dependence, which could be a therapeutic target [21]. Glutaminase inhibition also sensitized pancreatic cancer cells to gemcitabine, the conventional chemotherapeutic agent [16]. The current study confirmed these studies suggesting glutaminase inhibition could be a novel therapeutic approach. In addition to combination therapy with a conventional chemotherapeutic agent, metformin—an antidiabetic agent affecting glucose metabolism—has been tested in pancreatic cancer in combination with glutaminase inhibitor [20]. Together with glutamine metabolism, glucose metabolism will be an important target. Other Nrf2 activators, such as sulforaphane and dimethyl fumarate, are now undergoing clinical trials [22] and are available for clinical use [23], respectively. We need to evaluate whether these agents also enhance the antitumor effects of glutaminase inhibitors in pancreatic cancer cells, both in vitro and in vivo. These approaches will provide novel therapeutic options for pancreatic cancer in the near future.

## 4. Materials and Methods

### 4.1. Materials

Anti-histone H3 antibody (4499) and peroxidase-conjugated anti-rabbit antibody (7074) were purchased from Cell Signaling Technology (Beverly, MA, USA). Anti-Nrf2 and anti-Keap1 antibodies have been described previously [24,25]. Peroxidase-conjugated anti-mouse antibody (NA931) was purchased from GE Healthcare (Piscataway, NJ, USA). Anti-Nqo1 antibody (ab2346) was purchased from Abcam (Cambridge, UK). Other reagents were purchased from Sigma-Aldrich (St. Louis, MO, USA), unless otherwise specified.

### 4.2. Mice

*Nrf2^−/−^* and *Keap1^fl/fl^* mice have been described previously [26,27]. *Pdx-1-Cre*, *K-ras^LSL-G12D/+^*, and *p53^LSL-R172H/+^* mice were obtained from the NCI Mouse Repository (Frederick, MD, USA) [28]. These mice were crossed to obtain the following mice: *Pdx-1-Cre::K-ras^LSL-G12D/+^::p53^LSL-R172H/+^::Keap1^fl/fl^::Nrf2^+/−^* (KPC::K0N1) and *Pdx-1-Cre::K-ras^LSL-G12D/+^::p53^LSL-R172H/+^::Keap1^fl/fl^::Nrf2^−/−^* (KPC::K0N0). The mice were fed standard chow and water ad libitum. They were treated according to the Guidelines for Proper Conduct of Animal Experiments proposed by the Ministry of Education, Culture, Sports, Science, and Technology of Japan. This study was approved by the Institutional Animal Care and Use Committee of the Tohoku University Environmental & Safety Committee (Article no. 2019MdA-154).

### 4.3. Cell Lines and Cell Culture

Panc-1, MIAPaCa-2, and BxPC3 cells (human pancreatic cancer cell lines) were purchased from ATCC (Manassas, VA, USA). KPC-mouse‒derived pancreatic cancer cell lines (KPC lines 1 and 2) and KPC::Nrf2^−^^/−^-mouse‒derived pancreatic cancer cell lines (KPCN lines 1 and 2) have been described previously [8]. Pancreatic cancer tissues from KPC::K0N0 mice and KPC::K0N1 mice were excised and washed with Hank’s balanced salt solution, followed by collagenase P treatment (Roche Applied Science, Mannheim, Germany) for 10 min (0.5 mg/mL, 37 °C). Recovered cells were seeded in Dulbecco’s modified Eagle’s medium supplemented with 10% fetal bovine serum. These cell lines were maintained at 37 °C in a humidified incubator in an atmosphere of 5% CO_2_.

### 4.4. RNA Extraction and Quantitative RT-PCR

Total RNA was extracted from each cell line using the RNeasy kit (Qiagen, Valencia, CA, USA). One microgram of RNA was subjected to cDNA synthesis using SuperScript VIRO Master Mix (Thermo Fisher Scientific, Waltham, MA, USA). The expression of genes of interest was quantified using the StepOnePlus real-time PCR system (Thermo Fisher Scientific) and Fast SYBR Green Master Mix (Thermo Fisher Scientific) with the following primers: *β-actin* (forward 5′-GGCTGTATTCCCCTCCATCG-3′, reverse 5′-CCAGTTGGTAACAATGCCATGT-3′) [28], *cytokeratin 19* (forward 5′-GGGGGTTCAGTACGCATTGG-3′, reverse 5′-GAGGACGAGGTCACGAAGC-3′) [29], and *Gstm1* (forward 5′-CTACCTTGCCCGAAAGCAC-3′, reverse 5′-ATGTCTGCACGGATCCTCTC-3′) [30].

### 4.5. Western Blot

For total cell lysates, cells were lysed in RIPA buffer. The nuclear fraction was prepared using NE-PER Nuclear and Cytoplasmic Extraction Reagents (Thermo Fisher Scientific). Lysates were electrophoresed using NuPAGE 8% Bis-Tris Gel (Thermo Fisher Scientific) and transfered onto Immobilon-P Membrane (Merck, Darmstadt, Germany). Membranes were incubated overnight at 4ºC with primary antibody (1:1000 dilution). After incubation with peroxidase-conjugated antibody (1:3000 dilution), reactive bands were detected using ECL Western blotting detection reagents (GE Healthcare).

### 4.6. Cell Growth Assay

Cells were seeded in 96-well plate at 5000 cells/well density. After 24 h of incubation, cellular growth was assessed by bromodeoxyuridine (BrdU) incorporation assay by using cell proliferation ELISA (Sigma-Aldrich) according to the manufacture’s protocol.

### 4.7. Cell Viability Assay

Cells were seeded in 96-well plates (5000 cells/well). Cells were treated with 100 μM DEM for 24 h, followed by treatment with 0°−10 μM of CB-839 or BPTES for 48 h. Cell viability was measured using the 3-(4,5-dimethylthiazol-2-yl)-2,5-diphenyltetrazolium bromide (MTT) assay. After incubating cells with 5 μg/mL MTT for 2 h, dimethyl sulfoxide was added (50 μL/well). The optical density was measured using a spectrophotometer (SPECTRA Max 190, Molecular Devices, San Jose, CA, USA) at a wavelength of 570 nm, with a reference wavelength of 690 nm.

### 4.8. Statistical Analysis

Statistical analysis was performed using the JMP Pro 15 software (SAS Institute Inc. Cary, NC, USA). The differences between more than two groups were analyzed using the Tukey–Kramer method. *p* < 0.05 was considered significant. The error bars show standard deviations.

## Data Availability

Detailed experimental methods are available upon requests.

## Supplementary Materials

The following are available online at https://www.mdpi.com/1422-0067/22/4/1870/s1.
