# Supplementary figures and images for: Nrf2 Activation Sensitizes K-Ras Mutant Pancreatic Cancer Cells to Glutaminase Inhibition
Source: Int J Mol Sci. 2021 Feb 14;22(4):1870. doi: 10.3390/ijms22041870 (PMC7918355; doi:10.3390/ijms22041870)

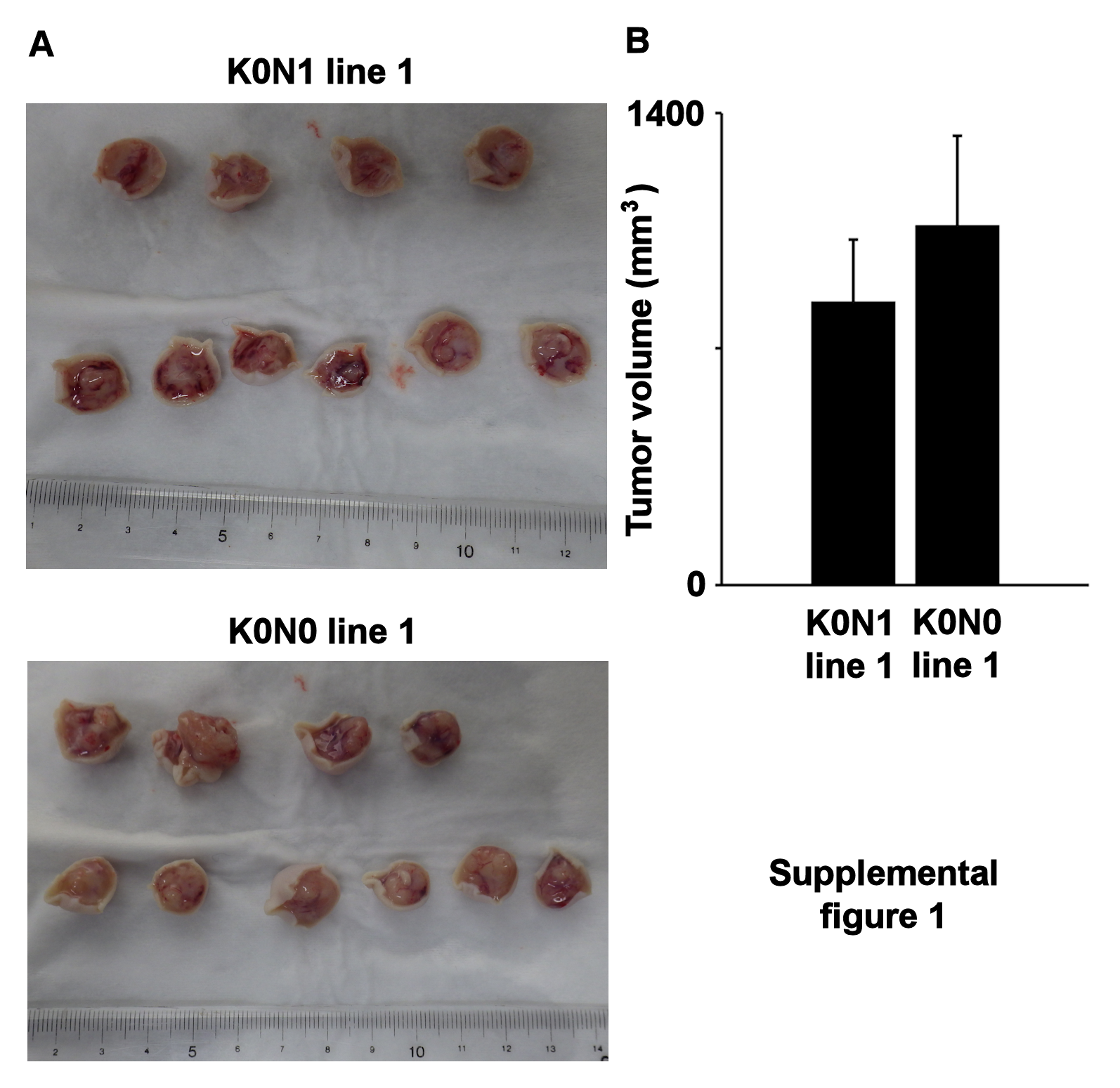

Supplement: Supplementary file 1 [file ijms-22-01870-s001.zip › ijms-1104613-supplementary/ijms-1104613-supplementary.tif]
